# MoO_3_-Doped MnCo_2_O_4_ Microspheres Consisting of Nanosheets: An Inexpensive Nanostructured Catalyst to Hydrolyze Ammonia Borane for Hydrogen Generation

**DOI:** 10.3390/nano9010021

**Published:** 2018-12-24

**Authors:** Dongsheng Lu, Yufa Feng, Zitian Ding, Jinyun Liao, Xibin Zhang, Hui-Ru Liu, Hao Li

**Affiliations:** School of Chemistry and Materials Engineering, Huizhou University, Huizhou 516007, China; dslu1018@163.com (D.L.); yufafeng@126.com (Y.F.); 13680800327@163.com (Z.D.); jyliao@126.com (J.L.); zxbin1@163.com (X.Z.); lhr@hzu.edu.cn (H.-R.L.)

**Keywords:** hydrogen production, ammonia borane, microspheres, nanosheets, heterogeneous catalysis

## Abstract

Production of hydrogen by catalytically hydrolyzing ammonia borane (AB) has attracted extensive attention in the field of catalysis and energy. However, it is still a challenge to develop a both inexpensive and active catalyst for AB hydrolysis. In this work, we designed a series of MoO_3_-doped MnCo_2_O_4_ (x) catalysts, which were fabricated by a hydrothermal process. The morphology, crystalline structure, and chemical components of the catalysts were systematically analyzed. The catalytic behavior of the catalyst in AB hydrolysis was investigated. Among these catalysts, MoO_3_-doped MnCo_2_O_4_ (0.10) microspheres composed of nanosheets exhibited the highest catalytic activity. The apparent activation energy is 34.24 kJ mol^−1^ and the corresponding turnover frequency is 26.4 mol_hydrogen_ min^−1^ mol_cat_^−1^. Taking into consideration the low cost and high performance, the MoO_3_-doped MnCo_2_O_4_ (0.10) microspheres composed of nanosheets represent a promising catalyst to hydrolyze AB for hydrogen production.

## 1. Introduction

With the rapid consumption of fossil fuels worldwide, environmental pollution is increasing, and the energy crisis is worsening. Therefore, seeking clean and sustainable energy to replace conventional fossil fuel energy is urgently required. Hydrogen, as a clean and sustainable energy carrier, has attracted widespread attention worldwide. It is considered part of a new generation of promising fuels due to its high energy density, zero emission, and easy availability [1,2]. However, the safe storage of hydrogen and its delivery represent bottlenecks that obstruct the commercializing of hydrogen energy. At present, hydrogen storage and transportation technologies mainly include hydrogen cryogenic liquefaction [3], pressurized tanks [4], and some hydrogen reversible adsorption materials, including carbon nanotubes and metal hydrides [5,6,7,8]. However, these approaches are either unsafe or expensive, which hinders their large-scale application. In recent years, hydrogen generation by hydrolyzing ammonia borane (AB) has caused extensive concern on account of the high hydrogen storage density (19.6 wt%) [9]. AB is hydrolyzed according to the following reaction (Equation (1)):

NH_3_BH_3_ + 2H_2_O → NH_4_BO_2_ + 3H_2_ (1)

Hydrolysis is the most common method for dehydrogenation of AB. However, at room temperature, AB does not hydrolyze by itself. Only by adding a suitable catalyst, can AB rapidly release a large amount of hydrogen. Therefore, in order to increase the hydrogen production rate, it is necessary to apply an inexpensive, stable, and efficient catalyst in the reaction from the kinetics perspective. In 2006, Xu et al. demonstrated for the first time that noble metals such as Pt, Rh and Pd exhibit high catalytic activity towards AB hydrolysis at room temperature [10]. Among these catalysts, the Pt catalyst has the highest activity and the best stability. Since then, a large number of noble metal catalysts were developed. Although noble metal-containing catalysts, such as Pd-PVB-TiO_2_ [11], Ag/Pd [12], Pd/graphene [13], RuPd@GO [14] and Pd [15] have exhibited high performance in this reaction, the high cost hinders their practical applications. In order to cut down costs, many researchers have incorporated non-precious metals into precious metals, such as Ru@Ni/C [16], Ag@Co/RGO [17], which have been used in AB hydrolysis. However, from a practical point of view, noble-metal-free catalysts with both high catalytic activity and low cost are more attractive. In recent years, many non-noble metal catalysts such as nickel, cobalt, iron and copper have been reported [18]. For example, Zahmakiran et al. prepared active carbon supported bimetallic copper-cobalt alloy by the surfactant-free deposition-reduction method and investigated its activity in AB hydrolysis [19]. Zhang et al. reported Ni nanoparticles supported on multi-walled carbon nanotubes by atomic layer deposition [20], which can also catalyze AB hydrolysis. Unfortunately, their catalytic activity is significantly lower than that of precious metal catalysts. Therefore, the development of new types of inexpensive catalysts with high performance for AB hydrolysis is still highly desirable.

In this study, we successfully prepared MoO_3_-doped MnCo_2_O_4_ microspheres comprised of nanosheets and investigated the material’s catalytic performance for the hydrolysis of AB. As far as we know, AB hydrolysis catalyzed by a MoO_3_-doped MnCo_2_O_4_ catalyst has not been reported yet. In addition, the dependence of the catalytic activity on the Mo doping amount was examined. It was determined that the catalytic performance of the catalyst can be improved by doping a proper amount of Mo.

## 2. Experimental Section

### 2.1. Synthesis of Catalysts

All reagents were of analytic grade. The preparation process of the MoO_3_-doped MnCo_2_O_4_ catalyst was as follows: At first, 2 mmol Co(CH_3_COOH)_2_·4H_2_O (Tianjin Kemiou Chemical Reagent Co.Ltd., Tianjin, China), 1 mmol Mn(CH_3_COO)_2_ (Tianjin Damao Chemical Reagent Factory, Tianjin, China), and 2 mmol CH_3_(CH_2_)_11_SO_4_Na (sodium dodecyl sulfate, SDS, Shantou Dahao Fine Chemicals Co.Ltd., Shantou, China) were dissolved in 20 mL of deionized water and were continuously stirred. Then, 24 mmol C_6_H_12_N_4_ (hexamethylenetetramine, Tianjin Damao Chemical Reagent Factory, Tianjin, China) was dissolved in 20 mL of deionized water, transferred to a sealing funnel, slowly added dropwise to the above-mentioned mixed solution and the mixture was vigorously stirred and mixed uniformly; subsequently, x mmol of ammonium molybdate (x = 0, 0.04, 0.10, 0.12; Tianjin ruijinte chemical limited companies) was added to the above solution, which was stirred for 30 min and transferred to a Teflon-lined stainless autoclave at 120 °C for 12 h; the precipitate was washed several times with deionized water and then dried in an electric vacuum drying oven at 60 °C for 2 h. The sample collected after drying was reacted at 350 °C for 2 h in a muffle furnace and the final products were labeled as MoO_3_-doped MnCo_2_O_4_ (x).

### 2.2. Characterizations

The crystalline structures of the samples were identified by X-ray diffraction (XRD) with a Rigaku D/Max-1200X diffractometer (Tokyo, Japan) employing Cu Kα radiation (40 kV, 200 mA and λ = 1.5406 Å). The morphology was analyzed using a Hitachi Su8010 scanning electron microscope (SEM, Hitachi, Japan). A Tecnai G2 F20 S-TWINT transmission electron microscope (FEI, Hillsboro, OR, USA) was applied to obtain TEM (Transmission Electron Microscope) and HRTEM (High- Resolution Transmission Electron Microscope) images. A Kratos Axis Ultra DLD X-ray photoelectron spectrometer (XPS, VG, Manchester, UK) was utilized to analyze the elements and oxidation states of the materials. The nitrogen adsorption-desorption isotherm and the Brunauer–Emmett–Teller (BET) surface areas of the products were determined using a 3H-2000 nitrogen adsorption apparatus (Quantachrome, Boynton Beach, FL, USA). The Fourier transform infrared spectrum (FT-IR) was measured using a TENSOR27 infrared spectrometer (Bruker, Karlsruhe, Germany), in which the powder sample was subjected to KBr tableting; the test spectrum ranged from 4000 to 400 cm^−1^.

### 2.3. Catalytic Experiments

In a typical test, 10.0 mg catalyst powder was dispersed in 5.0 mL water by an ultrasonication treatment in a conical flask, which was then placed into a water bath for maintaining the reaction temperature of 298 K. Subsequently, 3.0 mmol AB and 20.0 mmol NaOH was dissolved in 15 mL to form a mixed solution, which was added to the conical flask. The volume of the generated gas was determined by collecting it in a gas burette by water displacement.

## 3. Results and Discussion

**Figure 1:**
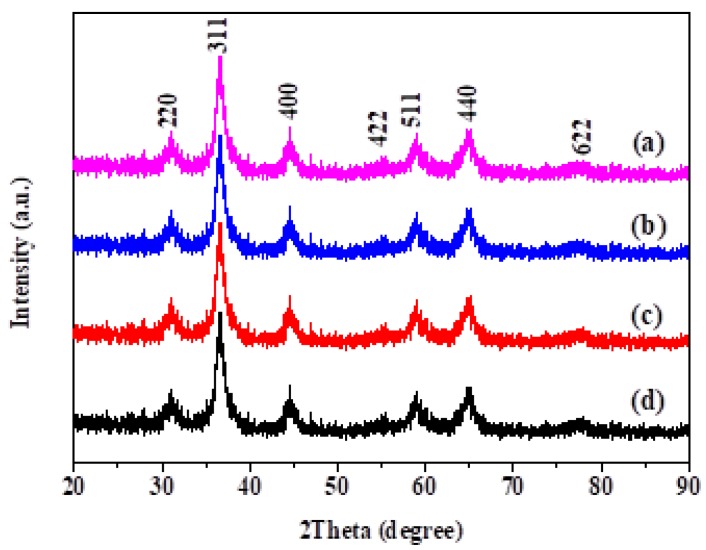
X-ray diffraction (XRD) patterns of the (**a**) MoO_3_-doped MnCo_2_O_4_ (0), (**b**) MoO_3_-doped MnCo_2_O_4_ (0.04), (**c**) MoO_3_-doped MnCo_2_O_4_ (0.10), and (**d**) MoO_3_-doped MnCo_2_O_4_ (0.12).

The XRD patterns of the MnCo_2_O_4_ with different amounts of doped Mo after the annealing treatment are shown in Figure 1. It can be concluded from the diffraction peaks that all of the four catalysts have MnCo_2_O_4_ cubic spinel structures (PDF No. 23-1237) and the space group is Fd3m [21]. Note that all the diffraction peaks are relatively wide, which may be due to a relatively low crystallinity of the material. No characteristic peaks corresponding to MoO_3_ are observed in the XRD patterns, which may result from the fact that MoO_3_ is amorphous [22].

**Figure 2:**
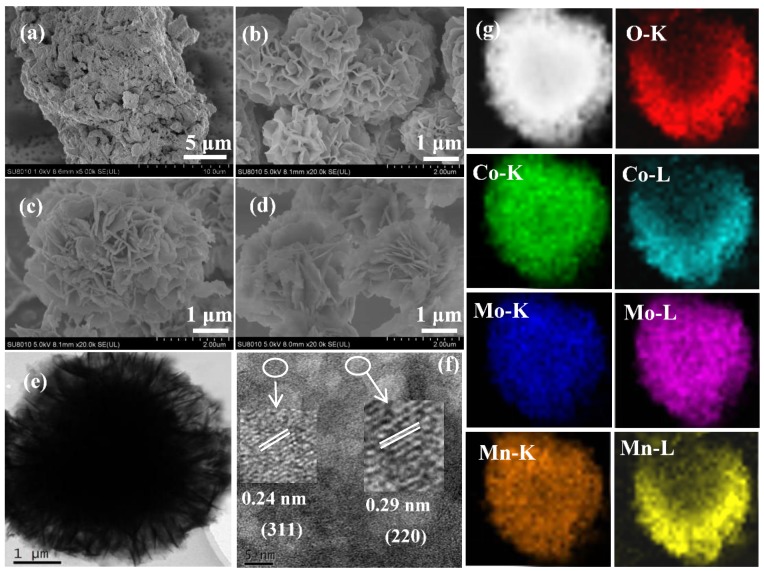
Scanning electron microscope (SEM) images of the (**a**) MoO_3_-doped MnCo_2_O_4_ (0), (**b**) MoO_3_-doped MnCo_2_O_4_ (0.04), (**c**) MoO_3_-doped MnCo_2_O_4_ (0.10), (**d**) MoO_3_-doped MnCo_2_O_4_ (0.12). (**e**) TEM image, (**f**) HRTEM image, and (**g**) elemental mapping images of the MoO_3_-doped MnCo_2_O_4_ (0.10).

**Figure 3:**
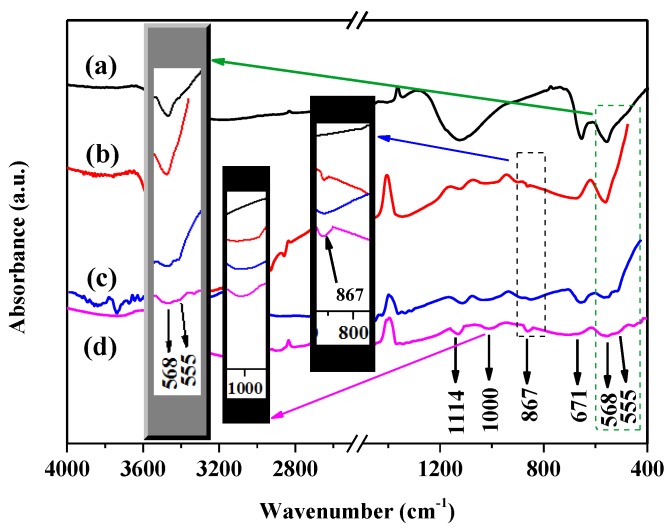
Fourier transform infrared spectrum (FT-IR) spectra of the (**a**) MoO_3_-doped MnCo_2_O_4_ (0), (**b**) MoO_3_-doped MnCo_2_O_4_ (0.04), (**c**) MoO_3_-doped MnCo_2_O_4_ (0.10), and (**d**) MoO_3_-doped MnCo_2_O_4_ (0.12).

SEM images of the MoO_3_-doped MnCo_2_O_4_ (x) are displayed in Figure 2a–d, in which microspheres are assembled from nanosheets. However, the MoO_3_-doped MnCo_2_O_4_ (0) consist of irregularly-shaped aggregation. Compared with the undoped MnCo_2_O_4_, all the MoO_3_-doped MnCo_2_O_4_ (x) catalysts have almost the same morphology, which are the microspheres with a typical size of 2–3 μm. To illustrate the effect of SDS in the synthesis, we carried out a contrast experiment, in which MoO_3_-doped MnCo_2_O_4_ (0.10) was synthesized in the absence of SDS while other conditions were kept unchanged. It was found that only some aggregated nanosheets were obtained without SDS (Figure S1 in the Supplementary Materials). This implies SDS plays an important role in helping the nanosheet to assemble into microspheres. Figure 2e displays a TEM image of MoO_3_-doped MnCo_2_O_4_ (0.10), which further confirms that the microspheres are composed of nanosheets. The high-resolution transmission electron microscopy (HRTEM) image of the samples (Figure 2f) reveals that the lattice fringes of the particles are 0.24 and 0.29 nm, which matches well respectively with the (311) and the (220) interplanar spacings of the MnCo_2_O_4_ [23]. The elemental mappings in Figure 3g shows that the Mn, Mo, and Co signals are observed in the microspheres, indicating that these three elements are uniformly distributed in the microspheres. A typical energy dispersive X-ray spectroscopy (EDX) pattern of MoO_3_-doped MnCo_2_O_4_ (0.10) sample is displayed in Figure S2 in the Supplementary Materials, in which the signals of elements of Co, Mn and Mo can be clearly seen.

To further verify the functional groups in the catalyst, we performed a FT-IR analysis of MoO_3_-doped MnCo_2_O_4_ (x). The absorption peak at 1114 cm^−1^ can be attributed to CO_3_^2−^. The absorption peak, CO_3_^2−^, may be caused by the absorption of CO_2_ from the air during the preparation process. The characteristic peaks at 671 cm^−1^ and 568 cm^−1^ can be indexed to the stretching vibration of the tetrahedral Mn–O and octahedral Co–O, respectively [24]. This further confirmed the spinel structure of the MnCo_2_O_4_ [25]. According to the literature, there are three peaks located at around 1000, 867 and 555 cm^−1^ in FT-IR spectrum of MoO_3_, which can be applied to identify MoO_3_. These three peaks are ascribed to the terminal oxygen symmetry stretching mode of Mo=O, the bridge oxygen asymmetry and symmetry stretching modes of Mo–O–Mo, respectively [26,27]. As shown in Figure 3, these three peaks can be seen in the FT-IR spectra of MoO_3_-doped MnCo_2_O_4_ (x), implying the presence of MoO_3_ in the samples. In order to further confirm the existence of MoO_3_ in MnCo_2_O_4_, Raman spectrum of the MoO_3_-doped MnCo_2_O_4_ (0.12) is displayed in Figure S3 in the Supplementary Materials. Evidently, three peaks related to MoO_3_ at 663, 818 and 995 cm^−1^ are observable, which is consistent with the results in the literature [28,29].

**Figure 4:**
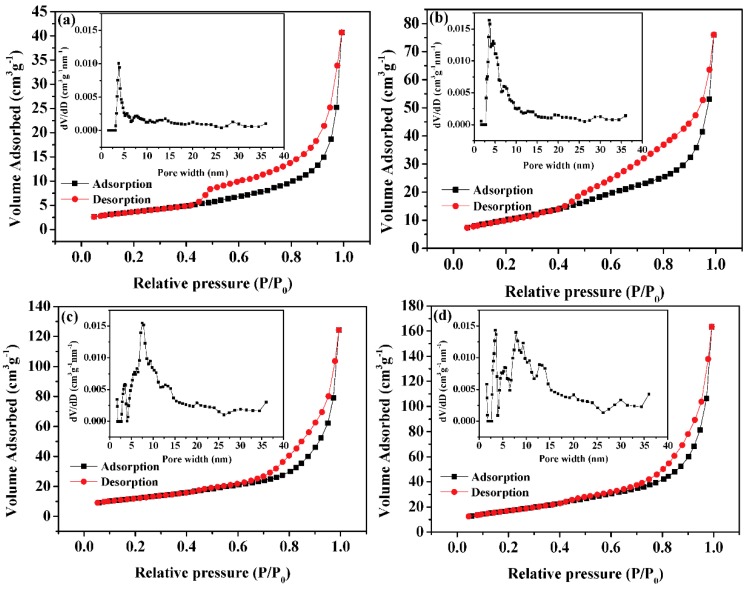
N_2_ absorption-desorption isotherms of (**a**) MoO_3_-doped MnCo_2_O_4_ (0), (**b**) MoO_3_-doped MnCo_2_O_4_ (0.04), (**c**) MoO_3_-doped MnCo_2_O_4_ (0.10), and (**d**) MoO_3_-doped MnCo_2_O_4_ (0.12) (inset: Pore size distribution).

Figure 4 shows the N_2_ adsorption-desorption curves of the four catalysts of MoO_3_-doped MnCo_2_O_4_ (x). The medium pressure section shows evidence of hysteresis. According to the classification by the International Union of Pure and Applied Chemistry (IUPAC), the isothermal adsorption curve is a typical Langmuir type IV curve, which represents multi-layer adsorption; there is no platform when the relative pressure value is high, indicating that there are many mesopores and macropores in the catalyst structure [30]. The inset in Figure 4 shows the corresponding pore size distribution. It can be found that with the increase in the amount of molybdenum, the pore size distribution becomes wide.

In many cases, the larger the surface area of a heterogeneous catalyst, the more active sites it may provide, therefore, the activity of the catalyst also increases. The surface area of the catalyst includes the inner surface and the outer surface. If the catalyst is non-porous, its surface area mainly comes from the outer surface. The smaller the particle size, the larger the specific surface area is. When the catalyst is porous, the surface area of the catalyst consists of inner and outer surfaces. The inner surface refers to the inner wall of the fine pores of the catalyst, whereas the remaining area is at the outer surface. In this case, the contribution of the inner surface area is fairly large in the total surface area, whereas that of the outer surface area is negligible. For our catalysts, with the increase of Mo content, the pore volume gradually increases, and the specific surface area of the catalyst increases correspondingly, which is consistent with the results shown in Table 1. Clearly, the doped Mo can prevent agglomeration and thus increase the BET surface areas of the catalysts. This is understandable because Mo in the form of oxide can serve as an atomic barrier to maintain the separation of nanostructure [31].

**Figure 5:**
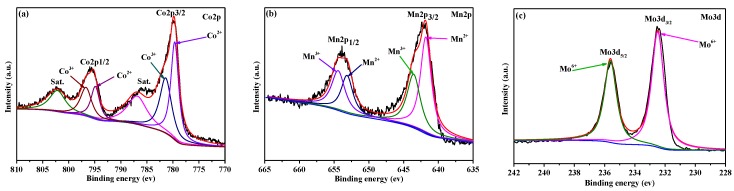
X-ray photoelectron spectrometer (XPS) spectra of (**a**) Co 2p, (**b**) Mn 2p, and (**c**) Mo 3d for the MoO_3_-doped MnCo_2_O_4_ (0.10).

XPS was used to investigate the oxidation state of the metal ions. Figure 5a is the Co 2p spectrum, in which two peaks were observable at 795.6 and 779.9 eV, indexing to Co 2p_1/2_ and Co 2p_3/2_, respectively. These two peaks could be decomposed into four peaks. The peaks at 779.6 and 794.9 eV was assigned to the Co^3+^ state. Meanwhile, the peak at 781.4 and 796.6 eV was ascribed to the Co^2+^ state in addition to two shake-up satellite (Sat.) peaks located at ca. 788.2 and 803.3 eV [32,33]. Similarly, in the Mn 2p spectrum (Figure 5b), the two peaks of Mn 2p_3/2_ at 642.0 eV and Mn 2p_1/2_ at 653.7 eV were divided into four separate bands: Two at 641.7 and 653.2 eV were assigned to the binding energy of Mn^2+^, whereas the other two at 643.5 and 654.2 eV were attributed to the existence of Mn^3+^ [34,35]. Figure 5c shows the XPS spectrum of the Mo 3d region. The two peaks at the binding energies of 934.4 and 954.2 eV were assigned to Mo 3d_3/2_ and Mo 3d_1/2_ of Mo^6+^ [36]. The molar content of Mo element on the catalyst surface is determined to be 6.65% with XPS. These results indicate that Co^2+^, Co^3+^, Mo^6+^, Mn^2+^ co-exist on the surface of MoO_3_-doped MnCo_2_O_4_ (0.10) microspheres, which are in good agreement with those in the literature for MnCo_2_O_4_ [37].

**Figure 6:**
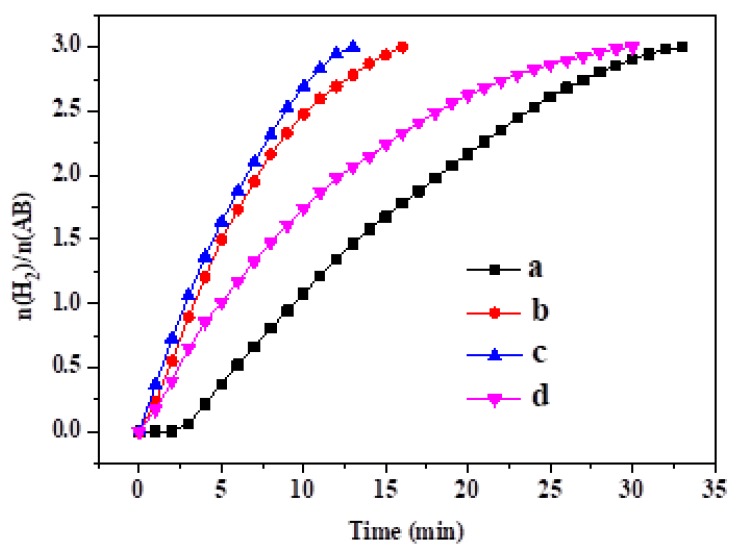
Hydrogen release from ammonia borane (AB) solution in the presence of (**a**) MoO_3_-doped MnCo_2_O_4_ (0), (**b**) MoO_3_-doped MnCo_2_O_4_ (0.04), (**c**) MoO_3_-doped MnCo_2_O_4_ (0.10), and (**d**) MoO_3_-doped MnCo_2_O_4_ (0.12).

Figure 6 shows the results of the catalytic activity tests of the MoO_3_-doped MnCo_2_O_4_ (x). It is observed that among the four catalysts, MoO_3_-doped MnCo_2_O_4_ (0.10) has the best activity and the hydrogen production rate is 22.5 mL min^−1^ at room temperature. The turnover frequency (TOF) reaches 26.4 mol_hydrogen_ min^−1^ mol_cat_^−1^. Note that AB hydrolysis is carried out in a basic solution in this work. For comparison, AB hydrolysis is carried out in a neutral solution. As shown in the Figure S4, hydrogen generation rate dropped sharply in the absence of NaOH. A similar result has also been reported by another group [38]. The following is also observed in Figure 6: As molybdenum is added, the hydrogen production is accelerated but an excessive doping amount of molybdenum has negative effects on the hydrolysis reaction. Three factors, including morphology, specific surface area and Mo content on the catalyst surface, should be taken into consideration when discussing the catalytic active sites of our catalysts. First, the un-doped MnCo_2_O_4_ is an irregularly-shaped aggregation. In contrast, MoO_3_-doped MnCo_2_O_4_ (x) is microspheres composed of nanosheets, which are expected to possess more corners and edges compared with the un-doped MnCo_2_O_4_ aggregation. Generally speaking, the atoms on the corners or edges have unsaturated valency with fewer bonds around them than those in the interiors or on the faces [39]. Thus, such atoms will display much higher intrinsic catalytic activity. Second, as shown in Table 1, compared with the un-doped MnCo_2_O_4_, the MoO_3_-doped MnCo_2_O_4_ (x) samples possess more pores and a larger BET surface, which will be favorable for the hydrolytic reaction of AB. Third, according to Fernandes et al., the adsorption of OH^−^ on the surface catalyst is an important step in AB hydrolysis and markedly affects the hydrolysis rate [31]. When Mo is doped into the sample, Mo^6+^ will act as a Lewis acid and facilitate the adsorption of OH^−^ on the catalyst surface, which enhances the hydrolysis reaction. However, an excessive amount of Mo in the sample is unfavorable for the AB hydrolysis, which may be attributed to the fact that an excess amount of Mo occupies the active sites on the catalyst surface. For comparison, we list the TOF values of some non-precious metal catalysts, as well as our catalyst in Table 2, and it is found that our catalyst exhibits a good activity.

**Figure 7:**
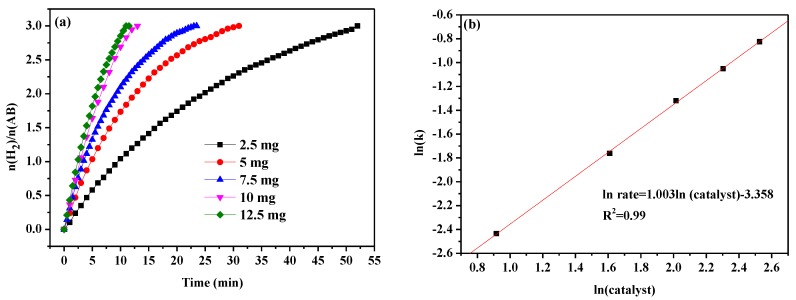
(**a**) AB hydrolysis with different dosage of MoO_3_-doped MnCo_2_O_4_ (0.10), and (**b**) the corresponding plot of ln(k) vs. ln(catalyst).

To investigate the reaction order of the catalysts, AB hydrolysis was carried out in the presence of various amounts of catalyst (2.5, 5, 7.5, 10, and 12.5 mg) and keeping all other conditions unchanged. As can be seen from Figure 7a, the higher the catalyst dosage, the faster the hydrogen generation is. The reaction order of the catalysts related to the hydrogen reaction can be calculated from the slope of the line in Figure 7b, which is 1.003. This demonstrating AB hydrolysis is a first order reaction for the catalyst. The observation is in a good agreement with other results reported in the literature [55].

**Figure 8:**
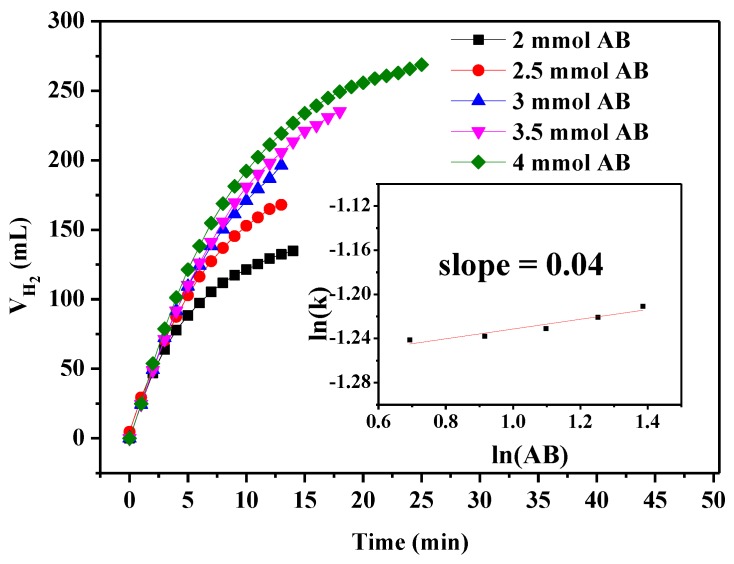
Hydrogen release at different concentrations of AB and the corresponding plot of ln(k) vs. ln(AB).

We also investigated the effect of different amounts of AB on the catalytic hydrogen production. As can be seen, the hydrogen production rates remain almost unchanged as the concentration of AB increases from 2 to 4 mmol (Figure 8), implying that the hydrogen generation rates are not affected by AB concentration. According to the line shown in the inset in Figure 8, the slope is 0.04, which is close to 0. Therefore, AB hydrolysis is a zero order reaction for AB [56].

**Figure 9:**
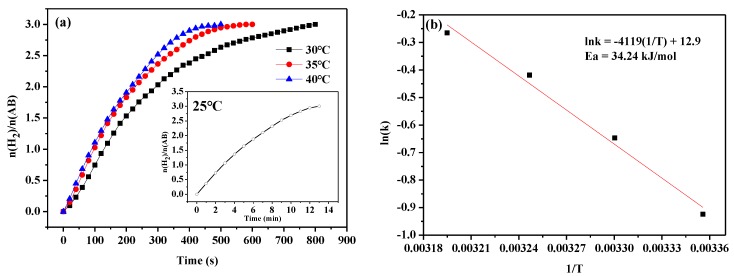
(**a**) Hydrolysis of AB at different temperatures catalyzed by MoO_3_-doped MnCo_2_O_4_ (0.10) and (**b**) the corresponding Arrhenius plot of ln(k) vs. (1/T).

Figure 9 shows the MoO_3_-doped MnCo_2_O_4_ (0.10) catalyst activity at different temperatures, indicating that the hydrogen release rate increases with an increase in the temperature. According to the Arrhenius equation, the apparent activation energy is Ea = 34.24 kJ mol^−1^ based on the data of the rate constants at different temperatures.

Based on these results, the kinetic equation for the MoO_3_-doped MnCo_2_O_4_ (0.10) catalyst can be deduced from the reaction order of the AB concentration and the catalyst concentration (Equation (2)):

(2) $$r=-\frac{1}{3}\frac{d[AB]}{dt}=k{\left[catalyst\right]}^{1.003}{\left[AB\right]}^{0.04}\approx k^{\prime }{\left[catalyst\right]}^{1.003},$$

(3) $$k^{\prime }=A\exp(-\frac{Ea}{RT})\Rightarrow \ln k^{\prime }=\ln A-\frac{Ea}{RT}.$$

The pre-exponential factor (A) is derived from the intercept of the illustration in Figure 9; the unit of A is mol s^−1^ g^−1^; k represents the rate constant (mol s^−1^ g^−1^), R is the molar gas constant (J K^−1^ mol^−1^), and Ea is the apparent activation energy (J mol^−1^). The following equation represents the rate law (Equation (4)):

(4) $$r=-\frac{1}{3}\frac{d(AB)}{d(t)}=400312\exp(-\frac{4119}{T})[catalyst].$$

## 4. Conclusions

In summary, the MoO_3_-doped MnCo_2_O_4_ microspheres consisting of nanosheets were successfully prepared by a hydrothermal synthesis reaction. The catalytic behavior of such nanostructured MoO_3_-doped MnCo_2_O_4_ in AB hydrolysis was studied. We discovered that the doping of molybdenum not only affected the final morphology of the product but also resulted in a small pore diameter, and an increase in the specific surface area. All these factors contributed to the enhancement of the catalytic activity. The optimum amount of molybdenum was determined using a performance test. This work can provide practical recommendations for the design and preparation of highly catalytic activity non-noble metal catalysts.

## Figures and Tables

**Table 1:** Brunauer–Emmett–Teller (BET) surface areas of different samples.

Catalyst	BET (m² g^−1^)	Pore Volume (cm^3^ g^−1^)	Pore Size (nm)
MoO_3_-doped MnCo_2_O_4_ (0)	13.24	0.069	3.81
MoO_3_-doped MnCo_2_O_4_ (0.04)	37.29	0.123	3.71
MoO_3_-doped MnCo_2_O_4_ (0.10)	43.26	0.193	3.48
MoO_3_-doped MnCo_2_O_4_ (0.12)	62.06	0.254	3.48

**Table 2:** Comparison the turnover frequency (TOF) of non-precious metal catalysts.

Catalysts	TOF (mol_hydrogen_min^−1^mol_cat_^−1^)	Reference
Co_0.79_B_0.15_P_0.06_/NGH	32.8	[40]
Cu-Co/PDDA-HNTs	30.8	[41]
Cu_0.49_Co_0.51_/C	28.7	[19]
MoO_3_-doped MnCo_2_O_4_ (0.10)	26.4	This work
Ni/CNTs	26.2	[20]
Cu_2_Ni_1_@MIL-101	20.9	[42]
CuCo@MIL-101	19.6	[43]
GeCH_3_	18.16	[44]
CoNi/Graphene	16.8	[45]
Cu_0.64_Ni_0.36_-TiO_2_	15.9	[46]
Co/Graphene	13.8	[47]
Ni_91_P_9_/rGO	13.3	[48]
b-CuO NA/CF	13.3	[49]
Co/NC-50	12.7	[50]
Ni_2_P	8.16	[51]
Cu/Co_3_O_4_	7.0	[52]
Ni/KB	5.9	[53]
Co@N-C	5.6	[54]

## Supplementary Materials

The following are available online at http://www.mdpi.com/2079-4991/9/1/21/s1, Figure S1: SEM image of the sample synthesized in the absence of SDS; Figure S2: EDX spectra of MoO_3_-doped MnCo_2_O_4_ (0.10) catalysts; Figure S3: Raman spectrum of the MoO_3_-doped MnCo_2_O_4_ (0.12); Figure S4: Hydrogen release from AB neutral solution in the presence of MoO_3_-doped MnCo_2_O_4_ (0.10) catalysts.
