# Correction: Lu, D., *et al*. MoO_3_-Doped MnCo_2_O_4_ Microspheres Consisting of Nanosheets: An Inexpensive Nanostructured Catalyst to Hydrolyze Ammonia Borane for Hydrogen Generation. *Nanomaterials* 2019, *9*, 21

**DOI:** 10.3390/nano9081112

**Published:** 2019-08-02

**Authors:** Dongsheng Lu, Yufa Feng, Zitian Ding, Jinyun Liao, Xibin Zhang, Hui-Ru Liu, Hao Li

**Affiliations:** School of Chemistry and Materials Engineering, Huizhou University, Huizhou 516007, China

In the original version of our article [1], Figure 1 included some incorrect data. The Figure 1 should be changed to the following figure:

The change has no material impact on the conclusions of our paper. The authors would like to apologize for any inconvenience caused to the readers by the change.

## Figures and Tables

**Figure 1:**
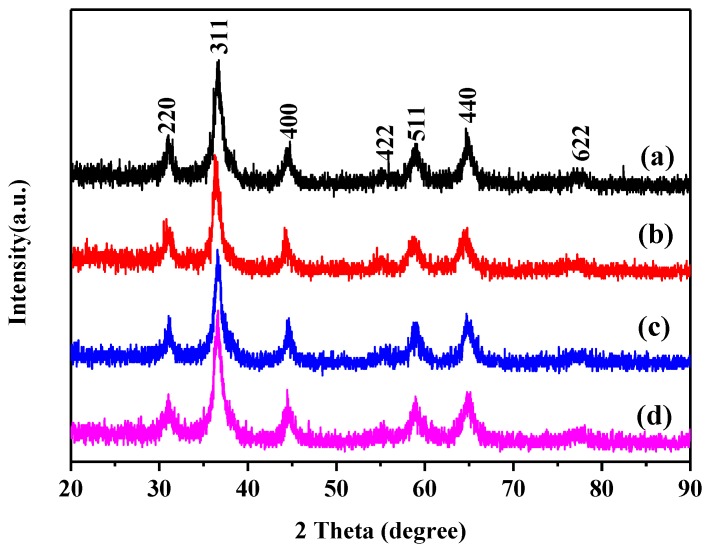
X-ray diffraction (XRD) patterns of the (**a**) MoO_3_-doped MnCo_2_O_4_ (0), (**b**) MoO_3_-doped MnCo_2_O_4_ (0.04), (**c**) MoO_3_-doped MnCo_2_O_4_ (0.10), and (**d**) MoO_3_-doped MnCo_2_O_4_ (0.12).
