# Supplementary material for: MoO3-Doped MnCo2O4 Microspheres Consisting of Nanosheets: An Inexpensive Nanostructured Catalyst to Hydrolyze Ammonia Borane for Hydrogen Generation
Source: Nanomaterials (Basel). 2018 Dec 24;9(1):21. doi: 10.3390/nano9010021 (PMC6359025; doi:10.3390/nano9010021)
Supplement: Supplementary file 1 [file nanomaterials-09-00021-s001.pdf]

Supplementary Materials

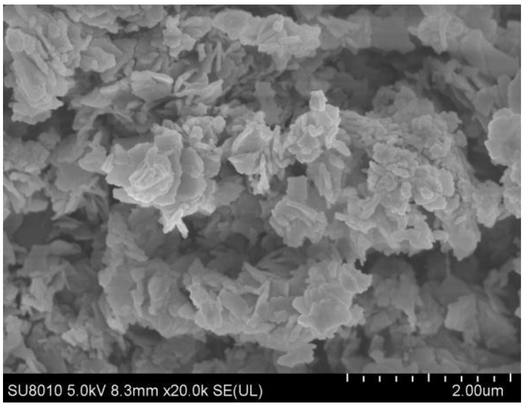

Figure S1. SEM image of the sample synthesized in the absence of SDS.

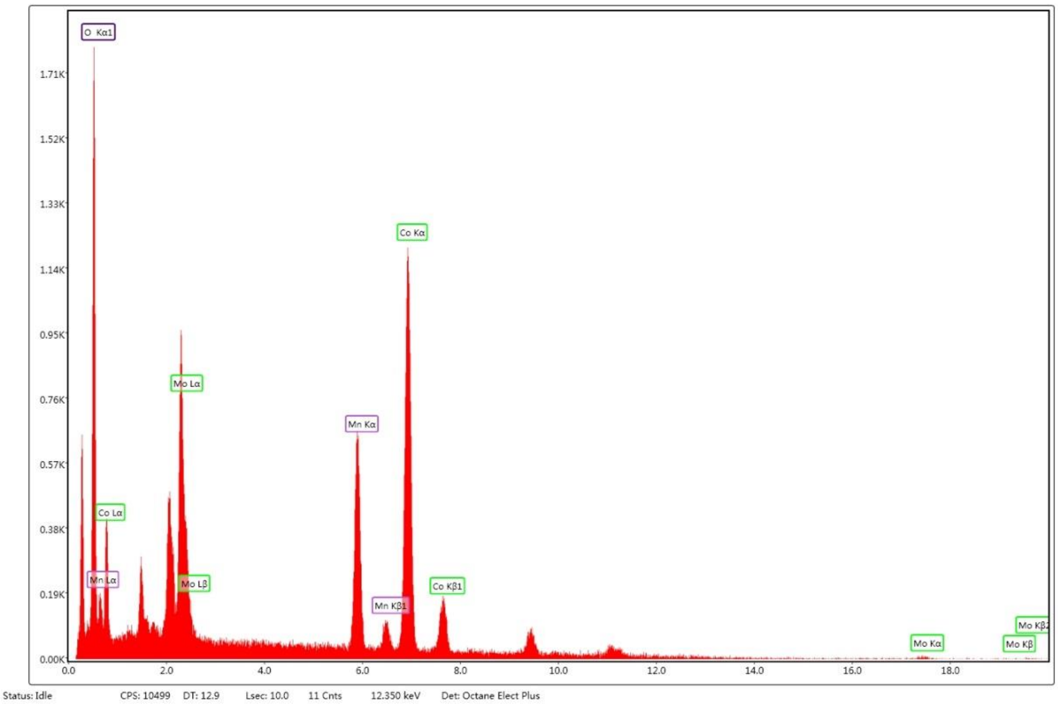

Figure S2. EDX spectra of MoO_3-doped $MnCo_2O_4$ (0.10) catalysts.

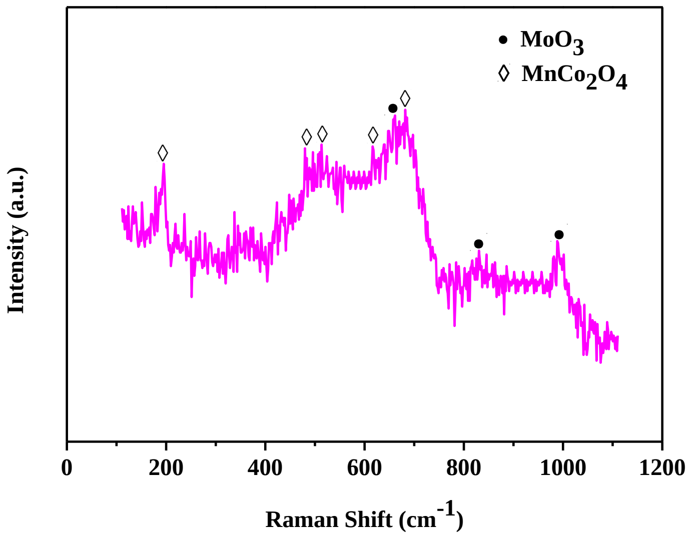

Figure S3. Raman spectrum of the MoO_3-doped $MnCo_2O_4$ (0.12).

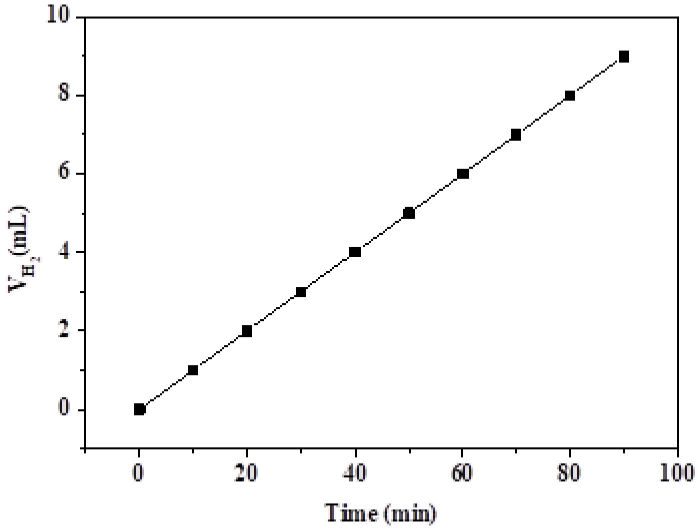

Figure S4. Hydrogen release from AB neutral solution in the presence of MoO_3-doped $MnCo_2O_4$ (0.10) catalysts.
